# Cyclooxygenase-1 (COX-1) and COX-1 Inhibitors in Cancer: A Review of Oncology and Medicinal Chemistry Literature

**DOI:** 10.3390/ph11040101

**Published:** 2018-10-11

**Authors:** Alessandra Pannunzio, Mauro Coluccia

**Affiliations:** Department of Pharmacy-Drug Sciences, University of Bari “Aldo Moro”, Via E. Orabona 4, I-70125 Bari, Italy; alessandra.pannunzio@uniba.it

**Keywords:** cyclooxygenase-1, cyclooxygenase-2, cancer, inflammation, tumorigenesis, COX-1 inhibitor

## Abstract

Prostaglandins and thromboxane are lipid signaling molecules deriving from arachidonic acid by the action of the cyclooxygenase isoenzymes COX-1 and COX-2. The role of cyclooxygenases (particularly COX-2) and prostaglandins (particularly PGE_2_) in cancer-related inflammation has been extensively investigated. In contrast, COX-1 has received less attention, although its expression increases in several human cancers and a pathogenetic role emerges from experimental models. COX-1 and COX-2 isoforms seem to operate in a coordinate manner in cancer pathophysiology, especially in the tumorigenesis process. However, in some cases, exemplified by the serous ovarian carcinoma, COX-1 plays a pivotal role, suggesting that other histopathological and molecular subtypes of cancer disease could share this feature. Importantly, the analysis of functional implications of COX-1-signaling, as well as of pharmacological action of COX-1-selective inhibitors, should not be restricted to the COX pathway and to the effects of prostaglandins already known for their ability of affecting the tumor phenotype. A knowledge-based choice of the most appropriate tumor cell models, and a major effort in investigating the COX-1 issue in the more general context of arachidonic acid metabolic network by using the systems biology approaches, should be strongly encouraged.

## 1. Introduction

Already in 1863, the German pathologist Rudolf C. Virchow had observed that some cancers were inherently associated with white blood cell infiltration [1]. However, it was only at the end of 20th century that an increasing body of research led to propose chronic inflammation as one of the enabling characteristics of cancer development [2,3], and the complex inflammatory network in premalignant and frankly malignant disease is now actively explored with the aim of translating epidemiological and experimental evidence into clinical practice [4].

Cancer may originate in the chronic inflammation setting associated with persistent infections, immune-mediated damage, or prolonged exposure to irritants. On the other hand, the genetic and epigenetic alterations underlying the cancerogenesis process inevitably modify the tissue homeostasis and may induce a chronic inflammatory response. Irrespective of the presumed primary or secondary nature of the process, inflammatory cells and mediators can be detected in most tumor tissues, where they act on both tumor and stromal cells and contribute to determine a tumor-promoting microenvironment [5].

In the general complexity of the inflammatory response (hundreds of chemical mediators have been identified, but how they function in a coordinated manner is still not fully understood), the arachidonic acid (AA) metabolites play a relevant role which appears intertwined with their functions in cell and tissue homeostasis [6]. Prostaglandins (PGs), including PGD_2_, PGE_2_, PGF_2a_, PGI_2_ and thromboxane (TX)A_2_ (collectively known also as prostanoids) are produced from arachidonic acid by sequential actions of cyclooxygenases (COX-1 or COX-2) and specific synthases, and they exert their effects in autocrine and/or paracrine manner mainly through G protein-coupled receptors (GPCRs) at the cell surface [7].

The involvement of prostaglandins in cancer was first evidenced in human esophageal carcinoma cells, when their invasive and metastatic potential in nude mice was found to be related to PGE_2_ and PGF_2a_ production [8]. Elevated levels of PGE_2_ have been found in numerous cancers, and its effects on multiple cell signaling pathways involved in tumor malignant phenotype induction and maintenance have been thereafter demonstrated [9,10,11,12,13]. However, PGE_2_ is not the only PG involved in carcinogenesis. PGD_2_ potentially contributes to the colon cancer risk in ulcerative colitis [14]; more recently, (TX)A_2_ was found to be involved in colorectal cancer pathophysiology [15,16], as well as in multiple myeloma [17] and lung [18] cancer cell proliferation.

The committed step of PG biosynthesis is catalyzed by COX-1 and COX-2 isoforms. These enzymes display many similarities in structure and catalytic properties and yield the same product, PGH_2_. However, COX-1 and COX-2 are different in their regulation of expression, tissue distribution, and associated synthases, thus subserving distinct biological tasks. COX-1 is constitutively expressed in most tissues (e.g., platelets, lung, prostate, brain, gastrointestinal tract, kidney, liver and spleen), where the COX-1-derived prostanoids are involved in homeostatic functions. It is generally accepted that COX-1 activity maintains the prostanoid production at a basal rate, and allows a rapid increase when cell membrane remodeling produces a rise of free AA. In contrast, COX-2 is generally considered as the inducible isoform, responsible for enhanced prostanoid production in response to inflammatory stimuli and growth factors during inflammation and various pathological conditions, including cancer [19,20]. It should be noted, however, that the concept of “constitutive” and “inducible” isoforms has been challenged by growing evidence indicating that both isoforms are present in normal tissues and can be up-regulated in various pathological conditions [21]. Both the expression and regulation of COX isoforms have been intensively investigated, and reviews about transcriptional regulatory mechanisms [22], and the regulation of gene expression at the co- or post-transcriptional level [23] have been recently published.

The role of cyclooxygenases (in particular COX-2) and prostaglandin products (in particular PGE_2_) in cancer-related inflammation has been extensively investigated in many neoplastic diseases, including esophageal [24], gastrointestinal [25,26] and pancreatic [27] cancers, breast [28] and cervical [29] cancers, renal [30], prostate [31], and bladder [32] cancers, skin [33,34] and head and neck [35] cancers, hematological tumors [36], and mesothelioma [37]. Tumor cells are often characterized by COX-2 aberrant expression [38,39], resulting from transcriptional and/or post-transcriptional alterations and contributing to tumor diseases, such as in colorectal cancer [40]. Importantly, the tumor-associated aberrant expression of COX-2 is often related to epigenetic alterations affecting COX-2 gene (PTGS2) as well as other genes involved into biosynthesis and signaling of its main prostaglandin products [41].

Epidemiological data are convincingly supported by various in vivo experimental systems, including chemically induced or transgenic models of colorectal, breast, and other types of cancer [42]. Finally, the therapeutic potential of aspirin, traditional nonsteroidal anti-inflammatory drugs (NSAIDs) and COX-2-selective inhibitors (coxibs) in human cancer has also been widely explored. A very large body of research, including about 2000 relevant publications only for aspirin, proposes that the anticancer activity of these drugs depends upon their ability to interfere with multiple pro-tumorigenic signaling pathways, some of them COX-dependent [38] and others COX-independent [43].

Unlike COX-2, the role of COX-1 in cancer has generally received less attention. Being usually considered as the constitutively expressed isoform that is involved in homeostatic cell and tissue functions, COX-1 was thought not be involved in carcinogenesis. However, increased levels of COX-1 expression have been occasionally reported in several cancers [44], and it has been shown that the genetic disruption of COX-1 is as effective as COX-2 disruption in reducing intestinal [45] and skin tumorigenesis [46] in mouse models, thus suggesting that both COX isoforms could cooperate in the cancerogenesis process. In the following, an overview of the available evidence about COX-1 expression and involvement in different neoplastic diseases will be presented. The objective of this review is to go through the COX-1-relevant literature in oncology and medicinal chemistry, thus attempting to fill the existing gap and highlighting the potential future studies.

## 2. COX-1 Involvement in Neoplastic Diseases

Renal cell carcinoma. The pathophysiological relevance of COX-2 in renal cell carcinoma (RCC) is generally acknowledged, and recently, it has been shown that COX-2 inhibition enhances the efficacy of immunotherapy and tyrosine kinase inhibitor-based treatment [30]. COX-1 expression, however, has been practically unexplored, except for a report on COX-1 mRNA overexpression in an RCC rat model [47]. Interestingly, two recent immunohistochemical investigations have shown a correlation between COX-1 overexpression and poor prognosis in RCC [48], and the validity of the combined use of COX-1 and VEGF in RCC histopathologic prognosis [49].

Skin cancer. The role of cyclooxygenase-dependent signaling in skin pathophysiology and non-melanoma carcinogenesis has been recently reviewed [33]. There is accumulating evidence that cyclooxygenase-2 may be involved in the pathogenesis of non-melanoma skin cancer, whereas COX-1 expression appears unaltered with respect to healthy tissue. At preclinical level, however, genetic studies show that the activity of both COX isoforms is mechanistically involved in the basal cell carcinoma pathogenesis [50,51].

Head and neck cancer. There are few data regarding COX-1 in head and neck cancer. A comparison of cyclooxygenase-1 expression levels between cancerous tissue from head and neck cancer patients and normal mucosa was performed by immunohistochemistry, Western blotting, and real-time RT-PCR (reverse transcription polymerase chain reaction), showing COX-1 overexpression in cancer cells and no expression in normal mucosa [52]. A progression-associated up-regulation of COX-1 expression was detected by immunohistochemistry in patients with hyperplasia, dysplasia, and carcinoma of oral mucosa [53] and a major expression of either COX-1 or COX-2 was reported in patients with oral squamous cell carcinoma with respect to normal mucosa [54]. More recently, tumor samples from patients with sebaceous gland carcinoma, an aggressive tumor commonly localized at Meibomian or Zeis glands, were examined by high-throughput tissue microarray for the expression of proteins involved in angiogenesis, inflammation, apoptosis, cell proliferation, cell-to-cell contact, and carcinogenesis. High expression of COX-1 and COX-2 was reported in 97% and 82% of patients, respectively [55]. Interesting implications on the COX-1 role derive also from in vitro investigations in head and neck squamous cell carcinoma (HNSCC) cell lines, in which the effects of pharmacologic inhibitors of cyclooxygenases were compared to those of small-interfering RNA as far as cell-growth, vascular endothelial growth factor (VEGF) production, and intracellular signaling are concerned [56,57]. The results showed that COX-2 inhibition blocked VEGF productions in some HNSCC cells; in contrast, other COX-2 (and PGE_2_) expressing HNSCC cells showed little response to COX-2 inhibition, this effect depending upon a differential expression of COX-1.

Esophageal cancer. Barrett’s esophagus, a complication of the chronic gastro-esophageal reflux disease, represents the best-known risk factor for esophageal adenocarcinoma development. Esophageal cancer prevention strategies [58], the role of inflammatory mediators in the disease pathophysiology [24], as well as encouraging experimental and epidemiological data on chemoprophylaxis with NSAIDs in patients with Barrett’s esophagus have been recently reviewed [59], along with a body of evidence supporting a role for COX-2 in the pathogenetic sequence leading to esophageal adenocarcinoma [60,61]. Interestingly, a possible involvement of COX-1 in the disease pathophysiology had already been suggested by the co-expression of both COX isoforms and angiogenic (VEGF-A) and lymphangiogenic (VEGF-C) growth factors in primary human tumor samples [62]. More recently, a possible cooperation of COX-1 and COX-2 isoforms has been suggested in an investigation of the PGE_2_ pathway in a rat model of esophageal adenocarcinoma induced by gastroduodenal reflux resulting from esophagojejunostomy [63], as well as by the finding that in the same experimental model indomethacin (a dual COX-1/COX-2 inhibitor) reduced the inflammatory lesions and tumor development, whereas a selective COX-2 inhibitor (MF-tricyclic) was ineffective [64]. Interestingly, a recent meta-analysis of nine observational studies has shown that both low-dose aspirin and non-aspirin COX inhibitor use is associated with a reduced risk of developing esophageal adenocarcinoma in patients with Barrett’s esophagus [65].

Colorectal cancer. In the colorectal carcinogenesis, the long-term transition process from normal mucosa to benign adenoma and final carcinoma provides the possibility to adopt preventive measures. Many evidences exist underlying the pathophysiological link between chronic inflammation and colorectal cancer [66], and the role of COX-2 in the carcinogenesis process has been extensively investigated [67,68]. COX-1 and COX-2 mRNA expression profiles were examined in tumor tissue in comparison to normal mucosa in stage III (Dukes’ C) colorectal cancer patients by Church et al. [69]. In contrast to the general opinion that constitutive COX-1 was not subject to variable expression, an altered regulation of COX-1 expression between normal and malignant tissues was reported, consistent with a COX-1 role in tumorigenesis. A significant correlation between levels of mRNA for COX-1, COX-2, TGF-beta1 and PGES, and those for proangiogenic factors VEGF-A and VEGF-C were also found in primary adenocarcinomas of the small intestine, thus suggesting a role for these factors in the propagation this rare neoplasia [70]. Interestingly, experimental mouse studies using genetic disruption of COX-1 or COX-2 plus *Apc* genes already 15 years ago suggested that both isoforms were involved in intestinal polyp formation [45]. Moreover, up-regulation of COX-1 protects intestinal stem cells from the DNA-damaging effect of azoxymethane and may play a key role in the early phase of intestinal tumorigenesis [71]. Furthermore, analysis of the expression levels of COX-1, COX-2, and mPGES (the downstream prostaglandin E synthase) in COX-1^−/−^ and COX-2^−/−^ ApcΔ716 double-knockout mice revealed that COX-1 was required from the early stage of intestinal polyp development, and that additional expression of COX-2 together with mPGES was necessary for subsequent accelerated growth of polyps [72]. On this basis, a mechanistic cooperation between COX-1 in the early stage of tumorigenesis and COX-2 in the subsequent polyp growth was proposed. In early stage of intestinal carcinogenesis, COX-1-PGE_2_ signaling associated with a suppression of the PG-catabolizing enzyme, 15-prostaglandin-dehydrogenase (15-PGDH), has been proposed to occur before COX-2 induction [73]. Recent studies have also shown that COX-1 is required for the maintenance of anchorage-independent growth ability of colon cancer cells (a key feature of malignant phenotype), as well as for tumor promoter-induced transformation of preneoplastic cells [74]. Interestingly, Li et al. in the same work identified a novel selective COX-1 inhibitor, 6-C-(E-phenylethenyl)-naringenin, a derivative of the flavonoid naringenin, also showing its chemopreventive efficacy in a colon cancer xenograft model.

Breast cancer. In the breast cancer microenvironment, there is a complex interplay between tumor and stromal cells, and a relevant contribute of COX-2-derived prostanoids is generally acknowledged [24,75,76]. However, both isoforms are expressed in breast cancer clinical samples, COX-1 being primarily localized in stromal cells [77,78]. Up-regulation of COX-1 gene expression in tumor tissues compared to normal tissue has been demonstrated also by whole genome expression analysis of breast carcinomas [79]. Moreover, induction of cell growth arrest and apoptosis are induced in MCF-7 human breast cell line in vitro by the COX-1 inhibitor FR122047 [80], and additive effects on tumor cell growth by COX-1 and COX-2 inhibitors are induced in vitro [81] and in vivo [82].

Cervical cancer. Human papilloma virus (HPV) infection of the cervical epithelium is well regarded as the main cause of cervical cancer [83], and a link between HPV E6 and E7 oncoproteins and COX-2 transcription has been established [84,85]. However, current data supporting a benefit for NSAIDs in the treatment of cervical intraepithelial neoplasia—the premalignant cervical lesion—are uncertain [86]. COX-1 up-regulation in cervical carcinoma was reported more than 10 years ago, and COX-1-dependent autocrine/paracrine regulation of COX-2, PGE_2_ receptors, and angiogenic factors was demonstrated in vitro [87]. Interestingly, it has been recently demonstrated that seminal plasma can promote cervical tumor cell growth in vitro and in vivo through the activation of inflammatory pathways involving both COX isoforms [88] and the expression of angiogenic chemokines [89]. Moreover, in an in vitro investigation of the co-regulation of COX-1 and downstream prostaglandin E synthases (mPGES-1, mPGES-2 and cPGES) in several tumor cell lines including cervical cancer, it was found that COX-1 and mPGES-1 messenger RNA (mRNA) are co-regulated and functionally coupled in basal PGE_2_ synthesis [90]. Although few clinical investigations of COX isoforms expression in cervical cancer are available [91,92], these experimental results, along with previous findings on the relationships between COX isoforms expression and radiosensitivity of cervical cancer cell lines [93] suggest that also the COX-1 role in cervical cancer prevention and treatment could be reevaluated [94].

Endometrial cancer. It is generally accepted that production of prostaglandins by endometrial epithelial cells under resting conditions is regulated through the constitutive expression of COX-1, whereas under stimulated conditions prostaglandin production is a result of the up-regulation of COX-2. A critical role for COX-2 isoform in the maintenance of endometrial tissue during the menstrual cycle as well as in the progression of endometrial cancer has been already observed [95,96], along with the potential clinical benefit of a selective COX-2 inhibitor in COX-2 positive endometrial cancers [97]. However, a possible involvement of COX-1 in the early stage of endometrial cancer development has been suggested, based on a higher COX-1 mRNA expression in patients with WHO (World Health Organization)-grade G1 and G2 endometrial cancer [98]. Moreover, a possible role for COX-1 in endometrial cancerogenesis has been suggested [99]. In a mechanistic investigation of the oxytocin ability of modulating the invasive properties of human endometrial carcinoma cells, the results showed that the hormone increased the invasive properties of tumor cells through the activation of PIK3/AKT pathway; this in turn led to up-regulation of both COX isoforms and subsequent PGE_2_ production. Interestingly, both COX isoforms were necessary and acted cooperatively for the oxytocin-induced invasion, COX-1 triggering the pro-invasive matrix metalloproteinase (MMP)-14 (a major MMP-2 activator), and COX-2 up-regulating MMP-2 expression. Importantly, an over-expression of oxytocin receptor was detected in endometrial cancer patients, thus indicating the clinical relevance of the oxytocin pathway in endometrial cancer progression.

Ovarian cancer. Epithelial ovarian cancer (EOC), the most lethal gynecological malignancy mainly occurring in older (postmenopausal) woman, is a highly heterogeneous disease [100], and includes distinct histological subtypes. A number of inflammatory cells and mediators are involved in EOC development and progression, and accumulating clinical and experimental evidence strongly suggests that inflammation might represent a unifying pathophysiological mechanism of ovarian carcinogenesis [101,102]. Interestingly, COX-1 was first identified as ovarian cancer marker two decades ago [103]. Since then, COX-1 over-expression has been reported by several groups in multiple human, mouse, as well as hen models of ovarian cancer [104,105,106,107,108,109]. COX-1 has been suggested as the major enzyme regulating PGE_2_ production in ovarian cancer cells [110], and along with COX-2 it plays an essential role in gonadotropin-induced tumor cell migration and invasion [111]. Thus, it is not surprising that both COX-1 and COX-2 have been included in a panel of inflammatory markers that characterize the rapidly growing and highly aggressive (type II) ovarian carcinomas [112]. Accordingly, the expression of COX-1 is higher in ovarian cancer patients with low CD8+ (cytotoxic T cells) and high CD1a+ (dendritic cells) cell density than in those with high CD8+ cell density [113], and COX-1 overexpression is the characterizing element in a cluster of ovarian cancer patients in which the poor prognosis is associated with an immunosuppressive status [114]. However, a role for COX-2 isoform in ovarian cancer has been also reported in clinical [115], as well as experimental investigations [116,117]. A major contribution for clarifying this apparent controversy comes from a very recent large-scale quantitative analysis of the expression of COX isoforms through The Cancer Genome Atlas (TCGA) dataset [118]. This study revealed markedly higher COX-1 expression than COX-2 in high-grade serous ovarian cancer (HGSOC)—the most aggressive EOC histotype—along with higher COX-1 expression in HGSOC tumors than 10 other tumor types in TCGA. Interestingly, a similar or higher expression of COX-2 isoform was instead observed for endometrioid, mucinous and clear cell tumors in an independent tissue microarray, thus suggesting a more relevant role for COX-2 in other EOC histotypes. Importantly, genetic knockdown of COX-1 in ovarian cancer cell lines resulted in down-regulation of both PG signaling and multiple pro-tumorigenic pathways, thus strongly encouraging further development of methods to selectively target COX-1 in the management of HGSOC tumors. The COX-1 relevance in ovarian cancer is also indirectly witnessed by the major efficacy of aspirin, a stronger COX-1 than COX-2 inhibitor, in COX-1-overexpressing in vitro and in vivo models of ovarian cancer [119,120,121,122].

Hematological tumors. COX-2 expression in hematological malignancies, including chronic lymphocytic leukemia, chronic myeloid leukemia, lymphoma, and multiple myeloma, favors tumor cell growth and survival, and represents a poor prognostic indicator [36]. As for myeloid and lymphoid acute leukemia, the transcription of both COX-1 and COX-2 isoforms in human leukemic blast cells from acute myeloid (AML) and acute lymphoid (ALL) patients has been well documented, but only COX-1 protein is expressed and active. COX-1-derived PGE_2_ stimulates the spontaneous growth of AML leukemic blasts in vitro [123]. Among the various COX-derived metabolites, only PGE_2_ appears to be endowed with a stimulating effect on the growth of leukemic blast cells in vitro [124], this suggesting a potential benefit stemming from COX-1 selective inhibition. COX-1, but not COX-2, is expressed and enzymatically active also in primary blasts from patients affected by acute promyelocytic leukemia (APL), a distinct AML subtype characterized by the t(15;17) translocation involving the PML gene on chromosome 15 and the retinoic acid receptor-alpha (RAR-alpha) gene on chromosome 17. A major component of APL therapy is all-*trans* retinoic acid (ATRA), based on its ability of activating gene transcription and tumor cell differentiation [125]. The ATRA-induced cell differentiation program is very complex, as shown by systems analysis of transcriptome and proteome [126], and includes also COX-1, but not COX-2, upregulation [127]. In this respect, it should be noted that the COX-1 selective inhibitor SC-560, as well as the non-selective indomethacin, impair both PGE_2_ production and ATRA-dependent differentiation of NB4 leukemic cells, a model of acute promyelocytic leukemia, thus suggesting that COX-1-inhibiting NSAIDs should be avoided in APL patients under ATRA treatment [128].

COX-1 and cancer stem cells. According to the cancer stem cell (CSC) model, tumor tissues are hierarchically organized as normal tissues, the cancer stem cells being able to fuel or reinitiate tumor growth, giving rise to the progeny which constitutes the bulk of tumor mass. The CSC niche homeostasis is regulated by a complex system of molecular signals, which include also the COX-derived AA metabolites [129,130]. In more detail, COX-2 is up-regulated in CSCs isolated from distinct tumor histotypes (e.g., in breast, colon and bone tumors), is co-expressed with stemness molecular markers, and promotes CSC growth in vitro systems, as recently reviewed by Pang L.Y. et al. [131]. This is not surprising, considering the relevance of COX-2-PGE_2_ system in stem cell biology of normal tissues [132], as well as the well-known relevance of COX-2 in cancer. However, some experimental evidence of COX-1 involvement in CSC biology also exists. In the azoxymethane (AOM) murine colon cancer model, the early molecular response of intestinal stem cells to genotoxic insult is driven by COX-1-PGE_2_ signaling, and results in increased stem cell survival [71]. In breast CSCs, isolated from primary cultures of spontaneous tumors from HER2/Neu transgenic mice, and characterized by proteomic analysis, both COX-1 and COX-2 genes are overexpressed with respect to non-CSCs. Moreover, both COX isoforms belong to an eight-gene signature that correlates with breast cancer patient survival, thus suggesting a role of both isoforms in breast cancers with HER2 overexpression [133]. More recently, the development of anticancer drug resistance has been demonstrated to depend also upon the activation of mesenchymal stem cells (MSCs), which are able of modulating in many ways the response to chemotherapy [134]. A peculiar occurrence after cisplatin treatment is the secretion of specific polyunsaturated fatty acids (12-oxo-5,8,10-heptadecatrienoic acid and hexadeca-4,7,10,13-tetraenoic acid) that are able to confer resistance to various anticancer drugs. Interestingly, the central enzymes involved in the synthesis of MSC-derived chemoprotective factors are COX-1 and thromboxane synthase [135], thus suggesting that enzyme inhibition could restore cancer cell sensitivity.

To summarize, there is a rich body of evidence suggesting that also COX-1 is involved in multiple aspects of cancer pathophysiology. In most cases, it seems that COX-1 and COX-2 isoforms operate in a coordinated manner, while in specific conditions a prominent role for COX-1 has been demonstrated. Functional consequences of COX-1 activation have been poorly investigated as compared to COX-2 activation, even though the effects of COX-1-PGE_2_ signaling on tumor cell phenotype have been described. However, a proper consideration should be given to the distinct functional modifications associated to COX-1 (or COX-2) expression [136], as shown also by a very recent genomic, lipidomic and metabolomic analysis of cyclooxygenase-null murine fibroblasts [137]. This study, which describes the common and dissimilar functional interactions of the COX isoforms at the cellular level by using a systems biology approach, demonstrates that COX-1 up-regulation results in a distinct “eicosanoid storm” along with an “anti-inflammatory, proinflammatory, and redox-activated” signatures. Even though fibroblasts are not cancer cells, these results suggest that COX-1 activity, when placed in pivotal position, can affect cell behavior even beyond eicosanoid metabolism.

## 3. Antitumor Activity of COX-1 Selective Inhibitors

**Figure 1:**
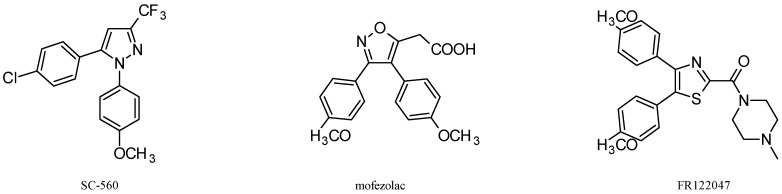
COX-1-selective inhibitors investigated for anticancer properties. SC-560,5-(4-chlorophenyl)-1-(4-methoxyphenyl)-3-(trifluoromethyl)pyrazole; mofezolac, (3,4-bis(4-methoxyphenyl)-5-isoxazolyl)acetic acid; FR122047, 1-((4,5-bis(4-methoxyphenyl)-2-thiazoyl)carbonyl)-4-methylpiperazine.

Specific inhibition of COX-2 has been extensively investigated, and many highly selective COX-2 inhibitors are available [138]. In contrast, relatively few COX-1-selective inhibitors have been described [139], and only some of them have been investigated for anticancer activity (Figure 1).

SC-560. SC-560 is a member of diaryl heterocycle class of COX inhibitors, discovered during the COX-2 inhibitor programs, and originally used as a pharmacologic tool to analyze the role of COX-1-derived prostaglandins in inflammation and pain [140]. The pharmacological action of SC-560 has been explored in various pathological conditions, and its antitumor activity has been investigated in multiple in vitro and in vivo experimental models of ovarian cancer and colorectal cancer, as well as other tumor histotypes, as described in the following.

Based upon a peculiar histopathological pattern showing elevated levels of COX-1 (not COX-2) in the epithelial compartment of ovarian tumor tissue undergoing extensive angiogenesis, the pharmacological action of SC-560 in ovarian cancer models was first investigated by Gupta R.A. et al. [104]. On ovarian cancer cell lines characterized for COX isoforms expression and activity, SC-560 showed in vitro antiproliferative effects similar to those of indomethacin and celecoxib (non-COX isoform selective and COX-2-selective, respectively), and only at doses 50–100 times greater than those achieved in in vivo systems [140]. In contrast, only SC-560 was found to inhibit arachidonic acid-induced VEGF secretion at low doses, an effect that was dose-dependently reverted by PGE_2_. The in vivo tumor growth inhibitory efficacy of SC-560 was then demonstrated by Daikoku T. et al. in genetically engineered ovarian cancer murine models, in which COX-1 overexpression was common to various disease-associated genetic alterations [105,106]. As far as COX-1 downstream targets are concerned, experimental evidence for PPARδ–ERK signaling involvement was provided in murine, as well as in human ovarian cancer [119]. The in vivo activity of SC-560 alone or in various combination protocols has been extensively investigated by Li W. et al. in the SKOV-3 xenograft model [141,142,143,144,145,146,147]. In this model, SKOV-3 cells predominantly express the COX-1 isoform, as determined by immunohistochemical analysis of tumor samples [142], and SC-560 treatment at COX-1-specific inhibitory dosages produced a slight to moderate reduction of tumor growth. Interestingly, combination treatment of SC-560 with cisplatin [147] or taxol [144,145,146,147] generally provided greater efficacy than individual agents on various pharmacological end-points, including angiogenesis, proliferation and apoptosis. Not surprisingly, a combination of SC-560 with the non-selective inhibitor ibuprofen [141] or the COX-2-selective celecoxib [143] also showed major efficacy in the SKOV-3 system, suggesting that the inhibition of both COX-1 and COX-2 could overcome a potential compensation between the two isoforms in the SKOV-3 xenograft biology. From a mechanistic point of view, the in vivo effects of SC-560 in the SKOV-3 system may be related to the treatment-dependent reduction of PGE_2_ production and to the associated inhibition of angiogenesis. However, a clear evidence for an exclusive or prevailing COX-1 dependence of prostaglandin production in SKOV-3 cells is lacking. Moreover, it cannot be excluded that in in vivo systems SC-560 could inhibit also COX-2 [148], and that COX-independent effects could contribute to antitumor activity. Some concern is partly due to the SKOV-3 cell line itself, which in some cases is reported as negative for COX-1 expression [104]. This is not actually unusual in the cyclooxygenase literature; the contrasting results variably depend upon a different source of the cell line, antibody cross-reactivity or other technical issues [149], and distinct experimental conditions (i.e., in vitro versus in vivo). Interestingly, SC-560 increases also paclitaxel sensitivity of taxane-resistant ovarian cancer cell lines characterized by MDR1/P-glycoprotein upregulation. A similar effect was produced by the COX-2 selective inhibitor NS398, not modified by PGE_2_ addition, thus suggesting a prostaglandin- and COX-independent mechanism [150]. The concrete possibility that SC-560 could represent a promising lead for ovarian cancer treatment is also suggested by the SC-560 belonging to a group of small molecule compounds that are potentially able to target ovarian cancer stem cell (OVCSC)-specific genes [151]. This interesting feature has been recently discovered through the characterization of OVCSC-specific gene expression profiling and a co-expression extrapolation with CMAP, a massive repository of gene expression data that provides information on gene expression modification of several cell lines treated with >1000 bioactive compounds [152].

In vitro and in vivo activity of SC-560 in comparison to celecoxib against colorectal cancer cells was investigated by Grosch S. et al. [153], by evaluating their effects on survival, cell cycle progression, and apoptosis of colon cancer cell characterized for COX-1 and COX-2 expression. Independently from COX status, both SC-560 and celecoxib affected in vitro cell survival and induced a G0/G1 block, whereas only celecoxib induced apoptosis. In vivo, both compounds were active towards a HCT-15 (COX-2 deficient) xenograft, but devoid of significant effect on HT-29 (COX-2 expressing) tumors, overall indicating a COX-independent mechanism of action. The effects of SC-560 on HT-29 colon cancer cells have been investigated also by Wu W. K. et al. [154], showing that the growth inhibitory effect was accompanied by G1-S transition arrest and phosphoinositide 3-kinase (PI3K)-induced autophagy. Proliferation inhibition and cell-cycle progression arrest induced in vitro by SC-560 has been investigated also on HCT-116 colon cancer cells, which had been previously characterized by a total absence of COX expression and activity [155]. Lee et al. showed that the growth inhibitory effect towards HCT-116 cells was affected by the cell cycle regulator protein p21CIP1 [156]. Moreover, Sakoguchi-Okada N. et al. showed that the growth inhibitory and apoptotic effect of SC-560 (and other non-selective and COX-2-selective inhibitors) on HCT-116 cells was associated with inhibition of survivin expression and Wnt/beta-catenin signaling pathway [157]. With the aim of identifying possible COX-independent mechanisms of action of NSAIDs, treatment-induced gene expression modifications of HCT-116 cells were investigated by using suppression subtractive hybridization [158]. Interestingly, two cancer-related genes, NAG-1 (officially known as GDF15), and thymosin β-4 (TMSB4X), were induced by SC-560, thus suggesting potentially contrasting effects on its antitumor activity. NAG-1 codes indeed for a pleiotropic cytokine of the TGF-β superfamily expressed in a broad range of cell types and involved in the cellular stress response program. NAG-1 induction effect may either be tumor-growth suppressive or enhancing, depending upon its induction pattern (acute or sustained) and pathophysiological context [159]. The induction of thymosin β-4 too could potentially compromise the anticancer activity of SC-560, being thymosin β-4 mechanistically involved in the metastatic process [160,161] and associated with poor prognosis in CRC [162]. Cyclooxygenase- and prostaglandin-independent growth inhibitory effects of SC-560 have been reported in vitro in cells representing various other tumor histotypes. The growth and cell-cycle progression of human A549, H460, and H358 lung cancer cells is affected by SC-560 at doses that are definitely higher than those required for COX-inhibiting activity. Interestingly, the growth suppression induced by SC-560, but not celecoxib treatment, was associated with reactive oxygen species production [163]. Multiple myeloma cell proliferation is also inhibited by SC-560 at doses that are 10 times higher than those necessary for enzymatic inhibition [164]. SC-560 treatment of HuH-6 and HA22T/VGH human hepatocellular carcinoma cell lines, expressing both COX isoforms at mRNA and protein level, led to growth inhibition and apoptosis, and inhibited anchorage-independent growth of HuH-6 cells in soft agar, an in vitro marker for malignancy of cancer cells [165]. From a mechanistic point of view, treatment of tumor cells with SC-560, as well as with the coxib CAY10404, was associated with activation of ERK1/2 signaling pathway.

Mofezolac. Among the few selective COX-1 inhibitors investigated as potential analgesics and anti-platelet agents, mofezolac has been developed and marketed in Japan as a powerful pain killer [166], and its anticancer activity has been investigated almost exclusively in colorectal cancer experimental models. In vitro treatment of a COX-1-expressing RGMI cell line (non-transformed cells derived from rat gastric mucosa) by mofezolac induces a weak apoptotic effect, substantially prostaglandin-synthesis independent, with respect to indomethacin or sodium diclofenac [167], and a similar behavior was also observed in AGS gastric adenocarcinoma cells, treated at concentrations that were similar to those found at gastric mucosa after oral administration [168]. With regard to in vivo models of colorectal cancer, mofezolac treatment suppresses the development of aberrant crypt foci (putative preneoplastic lesions) induced by azoxymethane (AOM) and reduces the number of intestinal polyps in Apc knockout mice. In both murine models, the efficacy of mofezolac was similar to that of nimesulide, this indicating a tumorigenic role for both cyclooxygenase isoforms in this experimental model [169]. Accordingly, a combination treatment with COX-1- and COX-2-selective inhibitors more effectively suppressed polyp growth than either of the single treatments alone in Apc knockout mice [170]. Besides inhibiting preneoplastic lesions, mofezolac significantly reduces the incidence, multiplicity and volume of azoxymethane-induced rat colon carcinomas [171], thus confirming the COX-1 pathophysiological role in the AOM-induced intestinal carcinogenesis. Interestingly, Mofezolac is also effective in suppressing beef tallow-promoted colon carcinogenesis in rats, thus suggesting a potential benefit for populations with high fat intake [172].

FR122047. The COX-1-selective inhibitor, 4,5-bis(4-methoxyphenyl)-2-[(1-methylpiperazin-4-yl)carbonyl]thiazole (FR122047) was originally developed as antiplatelet agent devoid of ulcerogenic effects [173], and investigated as analgesic agent [174], or used as tool for studying the involvement of COX-1 as well as the role of prostanoids generated along the COX-1 and COX-2 pathways in various models of inflammation [175,176]. The antitumor activity of FR122047 has been investigated in vitro on MCF-7 breast cancer cells [80]. FR122047 treatment inhibits in vitro cell growth of MCF-7 cells, and induces apoptotic cell death that is mechanistically independent from treatment-associated ROS production, as well as from PGE_2_ production inhibition. Mechanisms of MCF-7 cell death induced by FR12207 were further investigated by the same group [177], showing that FR122047 treatment induces caspase-mediated apoptosis and at the same time stimulates a defensive autophagic response of MCF-7 cells. Interestingly, the inhibition of caspase-9 blocks the cytoprotective autophagic process, thus increasing the susceptibility of MCF-7 cells to FR-122047-induced cell death.

In general, in vitro and in vivo antitumor properties of COX-1-selective inhibitors resemble those of other NSAIDs and coxibs; in some cases, COX-dependent, and in others, COX-independent mechanisms having been reported. Preliminary screening of the anticancer properties of COX inhibitors is usually performed by investigating the in vitro effect upon cell proliferation and cell cycle progression of tumor cells characterized for COX expression and activity. COX-1-selective inhibitors, not different from other NSAIDs or coxibs, impair cell growth and proliferation, this biological end-point being most often associated with cell cycle G1-phase arrest and cell death. These effects are usually observed at compound concentrations that are much higher than those of common cytotoxic drugs [178], and very often higher than clinically relevant COX-inhibitor concentrations. The true effective concentration of COX inhibitors in in vitro systems could actually be lower than that indicated, owing to a subtractive interaction with the serum proteins of culture medium [179]. However, whilst considering the protein interaction caveat, it is not surprising that COX-1 inhibitors (as well as traditional NSAIDs and coxibs) have weak antiproliferative effects related to enzymatic inhibition. This probably reflects the weak impact of COX-derived PGs in the maintenance of in vitro growth and proliferation condition. In the various inflammatory pathological conditions in which the PG activity has been investigated, these molecules do not operate as primary signals of homeostasis modification, but rather as amplification signals at the tissue level [19]. There is no reason to believe that in vitro tumor cell proliferation could be a different situation, as shown also by the lack of efficacy of COX inhibitors against resting tumor cells [180]. Therefore, to obtain more useful information from a basic antitumor assay such as cell growth and proliferation inhibition, it would be preferable to use tumor cells that increase their proliferation rate as a result of PG addition, and to demonstrate that this effect is impaired by the COX inhibitor under investigation. In this context, it would be preferable to integrate the COX expression and activity data into a more general knowledge of genes and gene products that are associated with AA metabolism in the tumor cells under investigation. This information is available to the scientific community in public databases, e.g., the Cancer Cell Line Encyclopedia [181], and it is essential for a reasoned choice of tumor cells to be used, also in relation to their suitability as models of human clinical disease [182]. The elegant paper of Wilson A.J. et al. [118] on the relevance of COX-1 in HGSOC witnesses the effectiveness of this strategy. Moreover, treatment effects upon cell cycle progression and the cell survival–death homeostasis could be more conveniently investigated in the same experimental setting by using modern multiparametric imaging technologies [183], thereby obtaining a comprehensive picture of treatment-associated phenotypic modifications. In in vitro systems again, specific investigations could be planned by using 3D tumor cell models enriched with stromal cells, to more closely mimic the tissue-like condition in which PGs usually operate. As far as other tumor phenotype hallmarks potentially affected by COX-derived PGs, such as angiogenesis, invasion and metastasis, and immune effects, they all would only be observed in in vivo systems, although some useful indication can be provided by in vitro assays measuring cell adhesion properties, migration, invasion, and colony formation [178]. As far as the COX-independent mechanism, which are often described for COX-1-selective inhibitors, they could also be of interest. Unlike what occurred with the COX-independent pharmacological actions of coxibs [184], which gave rise to substantial research and development activity [43], the results so far reported for COX-1-selective inhibitors have not triggered systematic research on this topic. This is not surprising, considering the small number COX-1-selective compounds available. On the other hand, a COX-independent mechanism occurs in an unpredictable manner, and sometimes in a different context, as in the case of the ability of mofezolac to interact with c-myc oncoprotein [185].

## 4. Conclusions

The involvement of COX-1 activity in cancer seems to have, in most cases, a pathophysiological role that is consistent and coordinated with COX-2, as in other inflammation-associated pathological conditions. However, in some cases, as exemplified by serous ovarian carcinoma, COX-1 overexpression plays a pivotal role, stimulating more in-depth studies on its function in this specific condition, and suggesting that other histopathological and molecular subtypes of cancer disease could share this feature. However, it seems clear that the analysis of functional implications of COX-1-signaling, as well as of the pharmacological action of COX-1-selective inhibitors, should not be restricted to the COX pathway, and to the effects of prostaglandins that are already known for their ability to affect the tumor phenotype. A knowledge-based choice of the most appropriate tumor cell models, and a major effort in investigating the COX-1 issue in the more general context of the arachidonic acid metabolic network by using systems biology approaches, should be strongly encouraged.
